# Effect of Deep Cryogenic Time on Martensite Multi-Level Microstructures and Mechanical Properties in AISI M35 High-Speed Steel

**DOI:** 10.3390/ma15196618

**Published:** 2022-09-23

**Authors:** Guili Xu, Peng Huang, Zhanhao Feng, Zhenxiong Wei, Guoyin Zu

**Affiliations:** School of Materials Science and Engineering, Northeastern University, Shenyang 110819, China

**Keywords:** AISI M35 high-speed steel, deep cryogenic treatment time, martensite multi-level microstructures, mechanical properties

## Abstract

High-speed steel is widely used for cutting tools due to its convenience of preparation and cost-effectiveness. Previous research has shown that deep cryogenic treatments improve the mechanical properties of high-speed steel, due to the transformation of the residual austenite and the precipitation of carbide, while few studies have researched martensitic changes. The variations in martensite multi-level microstructures in AISI M35 high-speed steel, treated over different deep cryogenic time periods, were investigated in this study. Meanwhile, the effect of these variations on the mechanical properties of the selected steel was discussed. It was found that prolonging deep cryogenic time facilitated an increase in dislocation, low-angle grain boundary, and the coincident-site lattice boundary (especially the twin boundary) of martensite. The size of the martensite block (*d_b_*) and lath (*d_l_*) decreased with deep cryogenic time. However, the effect on the microstructure was limited when the cryogenic treatment time exceeded 5 h. The increase in dislocation decreased the temperature for carbide precipitation and promoted fine carbide precipitation during tempering. The refinement of martensite multi-level microstructures and the greater precipitation of fine carbides gave the tempered specimens excellent impact toughness. The impact toughness of the tempered samples undergoing deep cryogenic treatment for more than 5 h was about 32% higher than the sample without deep cryogenic treatment.

## 1. Introduction

High-speed steel (HSS) was a widely used tool steel which is characterized by its high hardness, great wear resistance, superior impact toughness, and excellent cutting ability [1,2,3]. Deep cryogenic treatment (DCT) can further optimize the microstructure and enhance the mechanical properties of HSS to meet the efficiency and quality requirements of modern cutting [4,5,6,7,8,9,10]. As a supplementary process of heat treatment, DCT is widely used, environmentally friendly, and easy to operate [9,11,12]. According to the previous literature, DCT has a positive effect on the hardness [13], toughness [14], wear resistance [15], corrosion resistance [16], and service life [17,18] of HSS. M. Priyadarshini reported that the AISI P20 tool, when deep cryogenically treated for 9 h, increased 35% in micro-hardness hardness and 28% in wear resistance compared to the as-received untreated tool [19]. Düzce University showed that the AISI H11 steel, when deep cryogenically treated for 24 h, showed 14.5% less wear than the untreated tools [20]. It can be seen that DCT can improve the properties of steel, but the choices of DCT soaking time are various, according to different researchers.

As is well known, the matrix of HSS is martensite (M), which plays an important role in the mechanical properties of steel [21,22]. Martensite microstructures can be described via the descending levels of prior austenite grain (PAG), martensitic packet (MP), martensitic block (MB), and martensitic lath (ML) [23]. Each PAG is divided into several packets and the packet is then subdivided into the block with the same orientation; one block is composed of several laths [22]. Previous research has indicated that the toughness of the steel is enhanced with the decrease in the packet and block size under the conditions of the same prior austenite grain size [24,25]. Moreover, S.L. Long revealed that MB is the most effective control unit of strength, while ML is the most effective control unit of toughness in 20CrNi2Mo steel [26]. Martensite multi-level microstructures are one of the most important structures in martensitic steels.

However, most studies on the DCT of the HSS are mainly focused on the residual austenite (RA) stability and secondary carbide (SCs: 0.1–5.0 μm) precipitation [27,28]. There are few studies on the evolution and morphology changes of lath martensite with different DCT parameters. The relationship between the mechanical properties and martensite multi-level microstructures of the deep cryogenically treated HSS has not been revealed, which hinders the study of the toughening mechanism of DCT to the lath martensite microstructure and makes it difficult to predict the properties and microstructures according to DCT parameters. Therefore, the martensite multi-level microstructures of HSS with different DCT parameters needs further research.

This paper investigates the effect of different DCT processing times on the variation of the martensitic multi-level microstructures and the mechanical properties (including Vickers hardness and impact toughness) of the AISI M35 HSS. The relationship between microstructure evolution and mechanical properties is discussed. This work reveals the evolution of multi-level microstructures with DCT soaking time and provides some assistance in selecting the appropriate DCT soaking time.

## 2. Experimental Details

**Figure 1:**
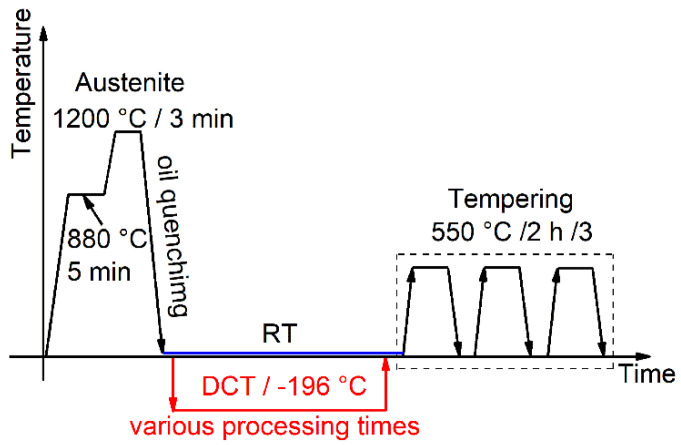
Schematic diagram of the heat treatment procedure.

The selected material, AISI M35, is one of the most common HSSs used for cutting tools (including helical and spline broaches, hobs, shaving cutters, and shaping cutters) [15,29]. The chemical compositions of AISI M35 (W6Mo5Cr4V2Co5) were measured using an optical emission spectrometer (OES), and the results are given in Table 1. Figure 1 shows the schematic diagram of the heat-treatment procedure. First, the steel was pretreated (880 °C/5 min) and then quenched (1200 °C/3 min) in oil to room temperature (RT). The specimen was fully austenitized to obtain a martensitic matrix. The quenched sample was kept at −196 °C (achieved by liquid nitrogen) for various pre-selected durations (x minutes/hours) before being heated back to RT. Finally, all samples (Q and D_3min_–D_48h_) were triple-tempered at 550 °C to ensure SCs precipitation. Details of the heat-treatment parameters are given in Table 2.

Microstructure characterizations were performed with an X-ray diffraction instrument (XRD, X Pertpro, Panaco, The Netherlands), a scanning electron microscope (SEM, ULTRA PLUS, Zeiss, Germany), electron backscatter diffraction (EBSD, Crossbeam550, Zeiss, Germany), and transmission electron microscopy (TEM, G20, FEI Tecnai, USA). The temperature of carbide precipitation was measured with a differential scanning calorimeter (DSC, 404F3, Nach, Germany). The XRD was conducted using an Ultima IV (Cu Ka radiation) at 40 kV, 40 mA, and 0.02° step size. The 2θ angular interval from 20° to 90° was step-scanned with 5°/min. The operating voltages for SEM and TEM were 15 kV and 200 kV, respectively. The EBSD experiments were conducted at a 25 kV and 0.15 μm step size.

The sample was electrolytically polished by the electrolyte, composed of alcohol (70 vol. %), perchloric acid (20 vol. %), and glycerol (10 vol. %) to analyze the distribution of SCs. The sample to be analyzed by OM and SEM was etched for ~60 s using nital, which is made up of concentrated nitric acid (4 vol. %) and ethyl alcohol (96 vol. %). An EBSD sample of 8 mm × 8 mm × 3 mm was polished by argon ion-beam milling to prevent deformation-induced transformation. The TEM specimen was prepared by grinding down from 400-μm to 50-μm-thick and slicing into 3-mm disks, then they were twin-jet electro-polished in a solution of 5 vol. % perchloric acid and 87.5 vol. % glacial acetic acid at 20 °C and 20–30 V.

In addition, a sample of 10 mm × 10 mm × 10 mm was used to measure the Vickers hardness. A sample of 10 mm × 10 mm × 55 mm was used to test the impact toughness with a V-notch. The hardness and impact toughness were performed at RT, and three samples were used in each group to reduce the possibility of test error.

The lattice strain (*ε_hkl_*) was analyzed by XRD data. The main calculation formulae are as follows [21,22]:

(1) $$\;d=\frac{n\lambda }{2sin\theta }$$

(2) $$\varepsilon_{hkl}=(d_{\left(hkl\right)}-d_{0})/d_{0}$$

where *d_(hkl)_* (Å) is the lattice space after heat treatments; *d*_0_ is the lattice space without deformation; *λ* is the wavelength of incident X-ray (*λ*_Cu_ = 0.15406 nm); n is the order of diffraction (*n* = 1). Martensite with a tetragonal structure meets the following:

(3) $$\frac{1}{a^{2}}(h^{2}+k^{2})+\frac{1}{c^{2}}l^{2}=\frac{1}{d_{\left(hkl\right)}^{2}}$$

where *a* and *c* are the lattice parameters. The tetragonality (*c*/*a*) can be calculated from Equations (1) and (3), after which the carbon concentration (*C_c_*) of M can be estimated by the following:

(4) $$C_{c}=\frac{\left(c/a-1\right)}{0.046}$$

SEM images of twenty randomly selected positions are studied using the Image-Pro Plus software, version 6.0. SCs with sizes of 0.1–0.5 μm (SSC), 0.5–1.0 μm (MSC), and 1.0–5.0 μm (LSC) are measured using different-magnification SEM images to reduce error [30]. However, the carbides with sizes of less than 0.1 μm could not be counted using SEM images but instead required TEM micrography.

## 3. Results

### 3.1. Mechanical Properties

**Figure 2:**
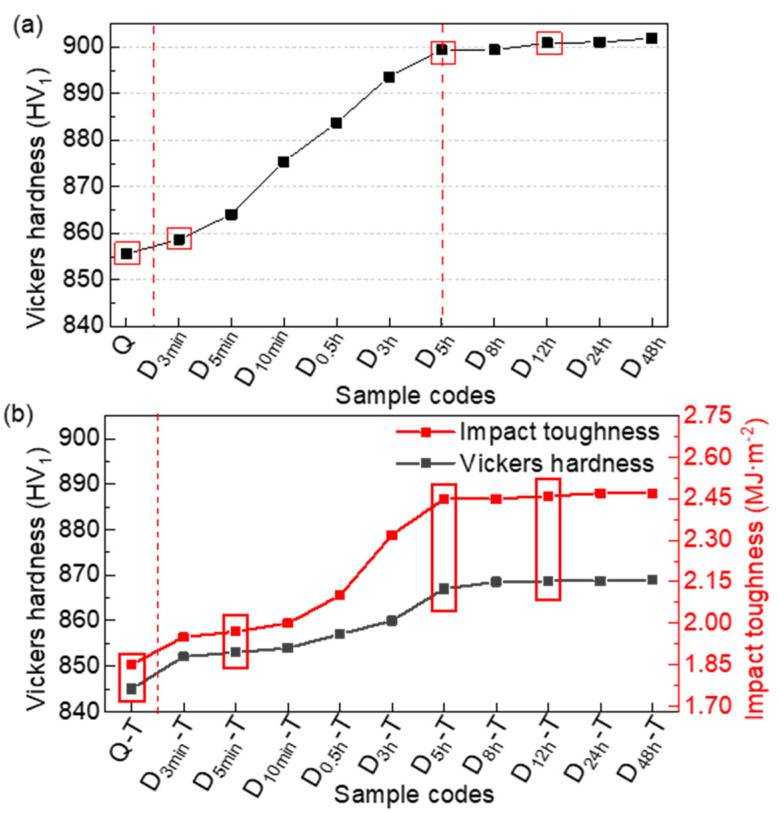
The mechanical properties of the (**a**) untempered and (**b**) tempered samples.

Figure 2a shows the average Vickers hardness of the samples, treated for different deep cryogenic durations. The hardness of the deep cryogenically treated samples (D_3min_–D_5h_) has a slight increase when the DCT processing time is below 5 h. However, the difference in the hardness is slight when the DCT soaking time is over 5 h. The deep cryogenic time has little effect on the hardness when it exceeds 5 h. The hardness of D_5h_, namely, ~899.38 HV_1_, increases by ~44 HV_1_ more than the quenched samples (Q), namely, 855.56 HV_1_. The studied samples in the DCT were selected from the initial section, the turning point, and the stabilization zone, according to the hardness variation. These selected samples were used together with the quenched samples to study the effect of cryogenic duration. The effect of DCT immersion time on the microstructures and mechanical properties was investigated via the Q, D_5min_, D_5h_, and D_12h_ samples (Figure 2a).

As shown in Figure 2b, the black line represents the average Vickers hardness of the tempered sample and the red line shows the average impact toughness of the tempered sample. Tempering reduces the hardness of the selected steel but obviously increases its toughness, resulting in better overall properties. The impact toughness and hardness of the tempered samples increase with DCT processing time, while the increase is slight when the DCT soaking time is higher than 5 h. The impact toughness and hardness of the D_5h_-T sample are 2.45 MJ/m^2^ and 867.05 HV_1_, respectively. The impact toughness of D_5h_-T is 32.4% higher than that of Q-T, and the hardness of D_5h_-Te increases by ~23.8 HV_1_ compared with Q-T (845.10 HV_1_). D_48h_-T has the highest value of impact toughness and hardness, which is 2.47 MJ/m^2^ and 868.90 HV_1_, respectively.

### 3.2. Microstructure Characteristics

**Figure 3:**
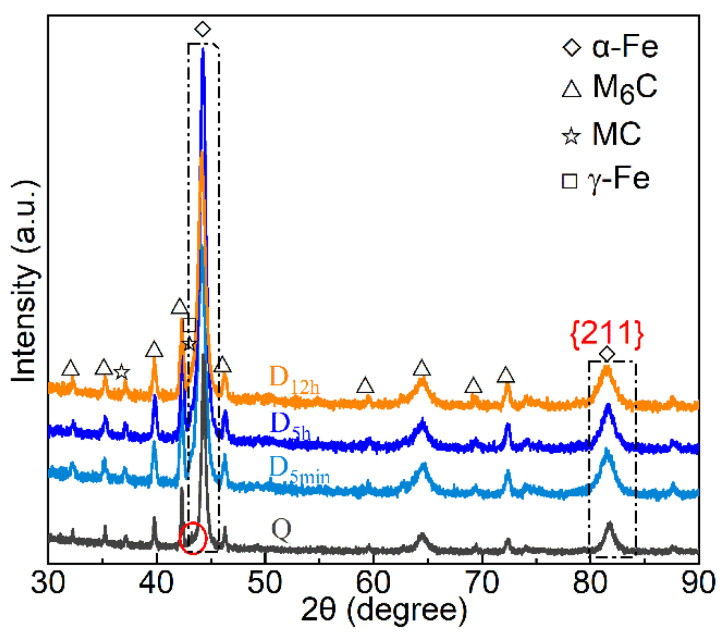
XRD pattern of Q, D_5min,_ D_5h_, and D_12h_.

**Figure 4:**
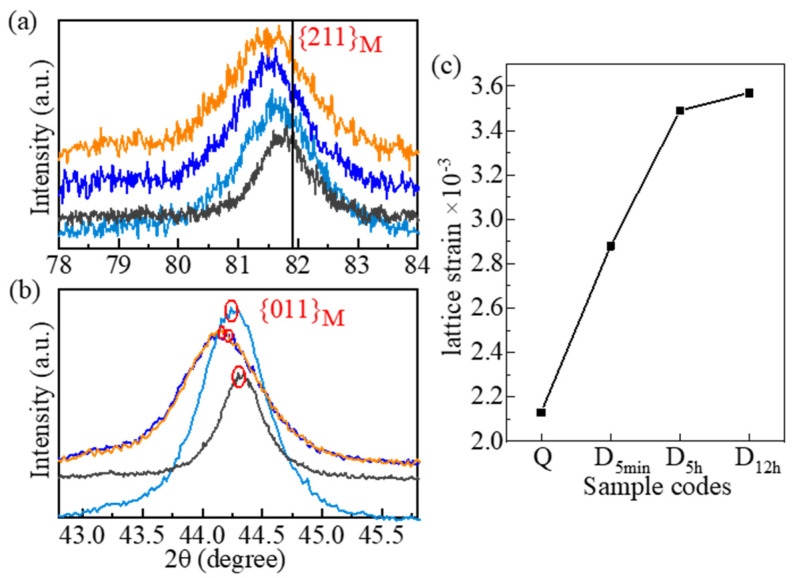
Enlarged view of (**a**) {211}_M_ and (**b**) {011}_M_ diffraction peaks, and (**c**) the lattice strain of {211}_M_.

Figure 3 shows the diffraction peaks corresponding to the M (α-Fe), M_6_C, and MC of samples subjected to different DCT soaking times [31,32]. The diffraction peaks of γ-Fe (RA) are present in the Q sample (red circle) but are absent in the DCT samples (D_5min_, D_5h_, and D_12h)_). Clearly, DCT promoted the decomposition of RA. Figure 4a,b show that the {211}_M_ and {110}_M_ diffraction peaks of all samples moved left, especially the DCT samples. The leftward movement of the DCT samples was more severe than that of the Q sample. The martensitic lattice space increased with DCT processing time. Moreover, the shift of the {211}_M_ and {011}_M_ diffraction peaks of D_5h_ and D_12h_ was more severe than that of D_5min_.

The {211}_M_ peak is used to calculate the lattice strain of M by Equations (1) and (2) since it is less disrupted by other peaks (Figure 4a). The lattice strain is shown in Figure 4c. The tetragonality (*c/a*) and carbon concentration (*C_c_*) are calculated from Equations (3) and (4) using the diffraction peaks of {011}_M_ and {211}_M_ (Figure 4a,b). The selection of {011}_M_ is the highest peak of M diffraction [33]. The value of *c/a* and *C_c_* are shown in Table 3.

Table 3 indicates that the value of *c/a* and *C_c_* increases with the DCT processing time. The M in the selected steel was subject to tensile stress due to the dissolution of high-alloying elements in the alloy (21.7 wt %) [34]. The tensile stress of M increased dramatically when the DCT processing time is from 0 to 5 h (Figure 4c). However, the tensile stress of M increased slightly when the DCT processing time lengthened from 5 h to 12 h. The values of *c/a* and *C_c_* in the D_5h_ and D_12h_ samples were similar.

**Figure 5:**
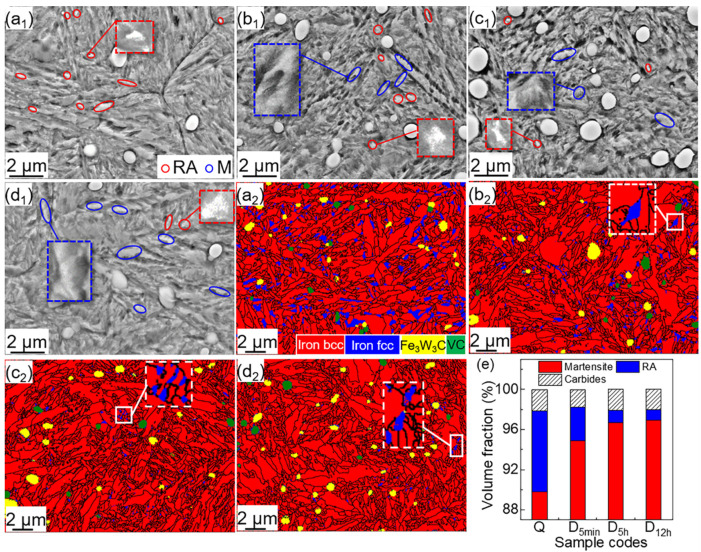
SEM micrographs and phase distribution maps of (**a_1_**,**a_2_**) Q, (**b_1_**,**b_2_**) D_5min_, (**c_1_**,**c_2_**) D_5h_, and (**d_1_**,**d_2_**) D_12h_, and (**e**) the volume percentage of the phases.

Although RA can be observed in the Q sample via XRD, its fraction in the deep cryogenically treated samples is less than the detection limits of XRD. Therefore, SEM and EBSD were adopted to further study the phase distribution and morphology of the Q, D_5min_, D_5h_, and D_12h_ samples. Figure 5a_1_–d_1_ show that all samples are composed of needle-shaped M, carbide, and a small amount of RA. Figure 5a_2_–d_2_ show that RA decreases with DCT processing time, and RA is hard to identify in the D_5h_ and D_12h_ samples_._ The volume fractions of RA in Q, D_5min_, and D_12h_ samples are ~8 vol %, ~3 vol %, and ~1 vol %, respectively (Figure 5e). The volume fraction of RA decreases when DCT processing time increases from 0 to 5 h, but it is similar in D_5h_ and D_12h_ samples at ~1 vol %. The increase in deep-cryogenic time has a limited effect on the decomposition of RA when the deep-cooling time exceeds 5 h.

### 3.3. Dislocation and Martensite Multi-Level Microstructure Size

**Figure 6:**
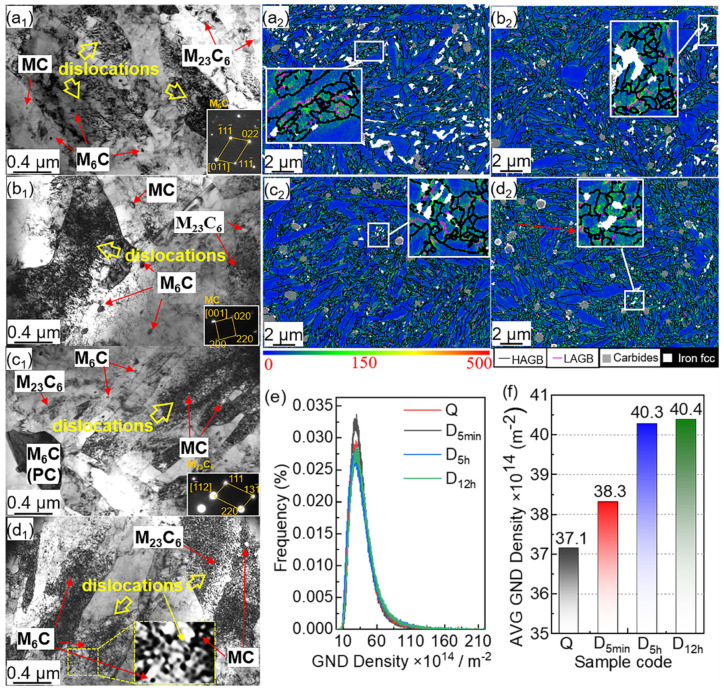
TEM micrographs and GND maps of (**a_1_**,**a_2_**) Q, (**b_1_**,**b_2_**) D_5min_, (**c_1_**,**c_2_**) D_5h_, and (**d_1_**,**d_2_**) D_12h_, and (**e**) the distribution and (**f**) proportion of GND density.

As shown in Figure 6a_1_–d_1_, many dislocations in the Q and DCT samples can be observed due to high lattice strain and plastic deformation during DCT. The dislocation increases with DCT time, and most fine carbides (M_6_C, MC, and M_23_C_6_) are present in the matrix of these sample_s_. D_5h_ and D_12h_ have the most dislocation and fine carbides. Meanwhile, the dislocation is entangled with carbides forming dislocation tangles, which enhance the material performance.

For measuring the dislocation details, geometrically necessary dislocation (GND) maps of these samples are presented in Figure 6a_2_–d_2_. High GND indicates a high-strain region. Figure 6a_2_–d_2_ show that the high-strain regions are concentrated at the martensitic boundaries, including the high-angle grain boundary (HAGB > 15°) and low-angle grain boundary (LAGB ≤ 15°). Moreover, the interface between M and RA phases represents the high-strain region. The distribution (Figure 6e) and average value (Figure 6f) of the GND density show that the average GND density increases with DCT processing time. In addition, the value of the average GND density in D_5min_ increases around 1.16 × 10^14^ m^−2^ compared to Q, while D_5h_ increases by 1.96 × 10^14^ m^−2^ compared to D_5min_. The value of average GND density in D_12h_ and D_5h_ is similar, at 40.4 × 10^14^ m^−2^ and 40.3 × 10^14^ m^−2^, respectively.

**Figure 7:**
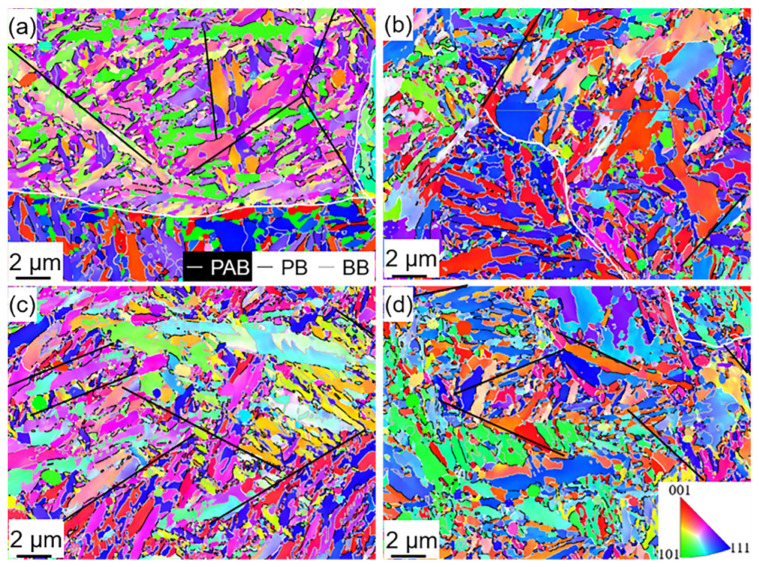
IPF (inverse pole figure) maps of (**a**) Q, (**b**) D_5min_, (**c**) D_5h_, and (**d**) D_12h_ (PAB—prior austenite boundary, PB—packet boundary, BB—block boundary).

**Figure 8:**
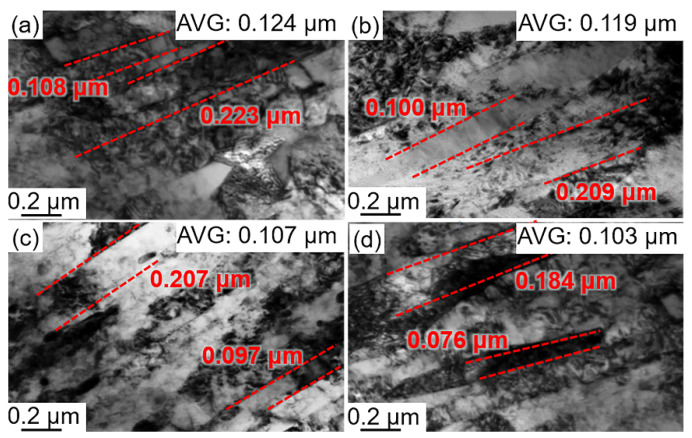
TEM micrographs of (**a**) Q, (**b**) D_5min_, (**c**) D_5h_, and (**d**) D_12h_ (AVG: average value).

Figure 7a–d show that the PAG is divided by several MP, which is subdivided into different MBs with parallel directions and different orientations. The MPs in the deep cryogenically treated samples were not analyzed because the effect of this low-temperature treatment on MP is negligible. The distribution of the martensite boundary orientation angles with the EBSD map is shown in Figure 8a. The MLs were observed via TEM, as shown in Figure 8a–d. The average diameter of the MBs and MLs was calculated from EBSD maps (Figure 7a–d) and TEM micrographs (Figure 8a–d), respectively. Table 4 shows that MBs and MLs are refined after DCT. The longer the DCT processing time, the more refined the martensite multi-level microstructures. The sizes of MBs (*d_b_*) in theD_5h_ and D_12h_ samples are 1.135 μm and 1.106 μm, respectively. The sizes of MLs (*d_l_*) in the D_5h_ D_12h_ samples are 0.124 μm and 0.119 μm, respectively. However, the value of *d_b_* and *d_l_* in D_5h_ and D_12h_ samples changes slightly. The *d_b_* in D_5h_ and D_12h_ samples are 1.028 μm and 1.021 μm, respectively. The *d_l_* in D_5h_ and D_12h_ samples are 0.107 μm and 0.103 μm, respectively.

**Figure 9:**
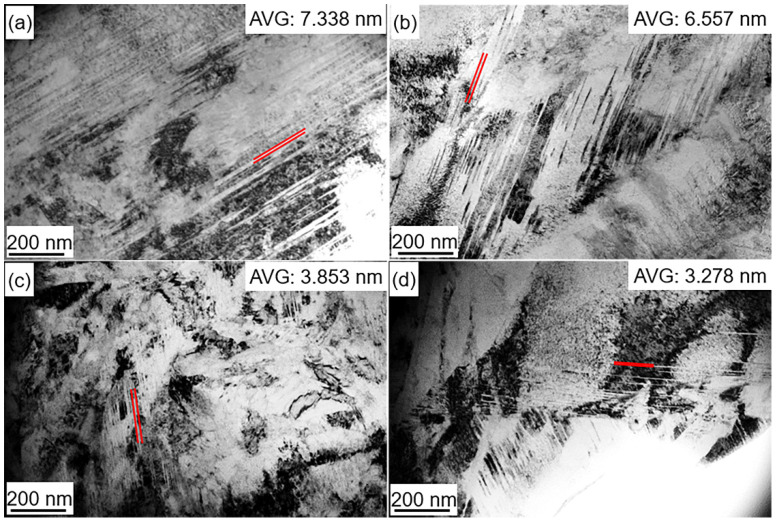
TEM micrographs of twin martensite in (**a**) Q, (**b**) D_5min_, (**c**) D_5h_, and (**d**) D_12h_ (AVG: average value).

Figure 9 shows that a large amount of twin martensite (TM) in Q and DCT specimens can be observed via TEM. The average sizes of the TM in Q, D_5min_, D_5h_, and D_12h_ samples are 7.338 nm, 6.557 nm, 3.853 nm, and 3.278 nm, respectively. Clearly, the TM is refined with DCT soaking time. The refinement is evident when the DCT treatment time goes from 0 to 5 h, but the TM sizes in D_5h_ and D_12h_ specimens are similar.

### 3.4. Martensite Boundaries

**Figure 10:**
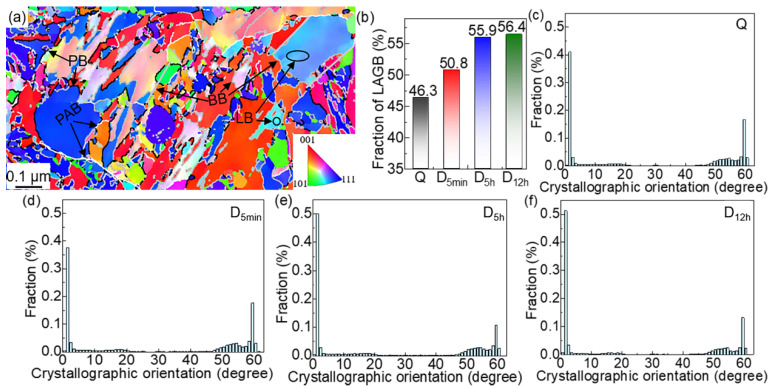
(**a**) The distribution of the martensitic boundary details, the (**b**) proportions, and (**c**–**f**) the distribution of LAGBs.

The grain boundary can be considered to be the LAGB and HAGB, based on the misorientation angle of the adjoining grains. LAGB is the ML boundary and HAGB is the boundary of the MB (55–65°), MP (45–55°), and PAG (15–45°). As shown in Figure 10c–f, LAGBs mainly concentrate in the range where the crystallographic orientation is less than or equal to 2°; the fraction of LAGBs in this range increases with DCT processing time. The LAGB fractions in Q, D_5min_, D_5h_, and D_12h_ samples are 46.3 vol %, 50.8 vol %, 55.9 vol %, and 56.4 vol %, respectively (Figure 10b). There is a dramatic increase in the LAGB fractions from Q to D_5h_ samples, yet there is only a little growth in the D_5h_ to D_12h_ samples. DCT promotes the production of LAGBs in M, and the fraction of LAGB increases slowly as the DCT soaking time reaches 5 h.

**Figure 11:**
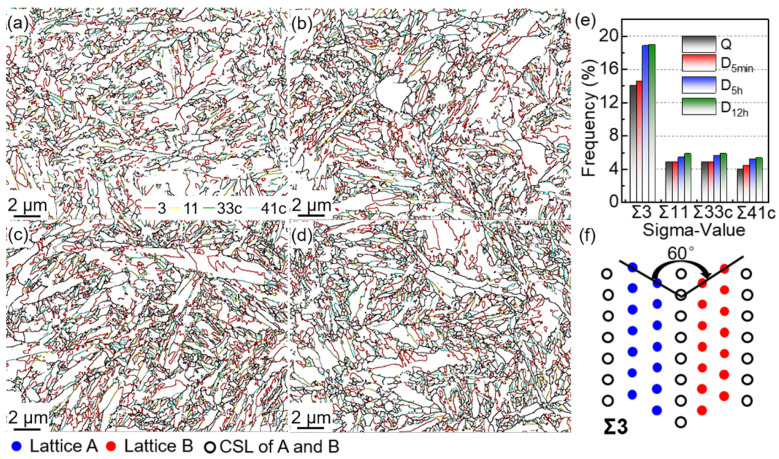
Martensitic CSL (coincident-site lattice) boundary maps in (**a**) Q, (**b**) D_5min_, (**c**) D_5h_, and (**d**) D_12h_, (**e**) the distribution of CSL boundaries, and (**f**) the crystals of Σ3.

Figure 11a–d give the M boundary maps combined with the CSL boundary. The general grain boundary is marked by black lines, while the coincident-site lattice (CSL) boundary is represented by colors (red, yellow, green, and blue). The CSL boundary is a special interface wherein the lattice satisfies the requirement of the new space lattice by sharing several lattice points [35]. The CSL boundary is categorized by Σ, which is defined as the inverse of common lattice points in the boundaries between the two adjoining grains or crystals. Figure 11a–d show that all samples have CSL boundaries, especially the DCT samples. Σ3 indicates that one of the crystals with the same dot structure is rotated by 60° relative to the other crystal, to the [111] crystal band axis (Figure 11f). Therefore, Σ3 is also the twin crystal boundary in M. The frequency of Σ3 in D_5h_ and D_12h_ samples (namely, ~19%) is dramatically higher than that in the Q (Figure 11e) sample. The increase in Σ3 represents the increase in fresh TM. It can be inferred that finer TM was formed during the DCT process, increasing the Σ3 boundaries (Figure 11) and refining the TM size (Figure 9).

Σ11, Σ33c, and Σ41c represent the crystals, which are rotated with the same dot structure by 50.48°, 58.98°, and 55.88°, relative to the other crystal in the [111] crystal band axis, respectively. As shown in Figure 11e, the frequency of other CSL boundaries in M (Σ11, Σ33c, and Σ41c) is much less than Σ3. Σ11, Σ33c, and Σ41c, which also increase with DCT time, but they show a lower rise than Σ3.

## 4. Discussion

### 4.1. Effect of Microstructures Evolution on SCs Precipitation

**Figure 12:**
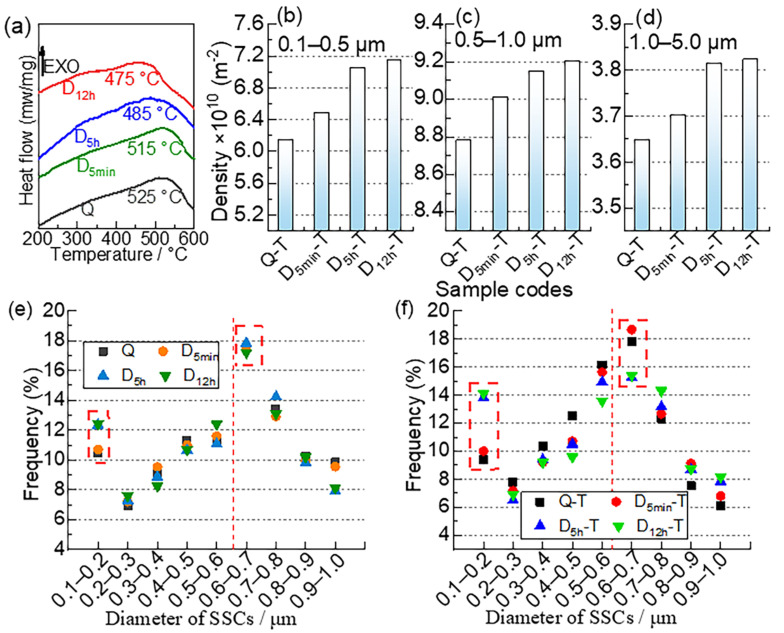
(**a**) DSC curves of Q and DCT samples, (**b**–**d**) the density of SCs, and the distribution of SCs in the (**e**) untempered and (**f**) tempered samples.

Precipitation strengthening is mainly based on the precipitation of SCs (M_6_C (M: Fe/W/Mo), MC (M: V/Fe), and M_23_C_6_ (M: Cr/Fe)) in AISI M35 HSS [21]. As shown in Figure 12e, the distribution of SCs (0.1–1 μm) in the Q and DCT samples is similar. The interstitial diffusion coefficient of carbon and the self-diffusion coefficient of iron both tend to zero at −196 °C [36], indicating few SCs precipitations.

Although DCT hinders the diffusion activity of atoms, the supersaturated carbon atoms in M are extruded out of the M lattice owing to the high lattice contraction of M under ultra-low temperatures (Figure 4c) [22]. The fraction of fine SSCs (0.1–0.2 μm) in the DCT samples is a little higher than the quenched sample and increases with DCT processing time (Figure 12e). Plenty of dislocations generated by high plastic deformation (Figure 6) and the fresh M transformed from RA (Figure 5) [34] are beneficial to the precipitation of fine SSCs [14,37]. Nevertheless, the *C_c_* increases after DCT, as shown in Table 3. On the one hand, the value of *C_c_* in fresh M is higher than in the virgin one. On the other hand, the DCT sample that was heated to room temperature led to the increase in “c” and the decrease in “a”, increasing the value of *C_c_* [33]. In addition, the fraction of SCs with a particle size of 0.6–0.7 μm in the untempered samples dominates the SCs (0.1–1.0 μm).

The carbide precipitation in HSS mainly occurs during the tempering process, due to the diffusion coefficient, which is affected by temperature. Figure 12a shows that the peak temperatures of carbide precipitation in the Q, D_5min_, D_5h_, and D_12h_ samples are 525 °C, 515 °C, 485 °C, and 475 °C, respectively. SCs will precipitate sufficiently during tempering since the tempering temperature (550 °C) is higher than the peak temperature of carbide precipitation in the Q and DCT samples. It can be seen that the temperature of carbide precipitation decreases with DCT process time. Fine carbides generated during DCT provide nucleation points for carbide precipitation during subsequent tempering (Figure 12e). Meanwhile, the increase in dislocation accelerates the diffusion process in the crystal, decreasing the temperature of carbide precipitation [38]. The dislocations increase with DCT soaking time (Figure 12e), so the peak of carbides precipitation declines accordingly.

Figure 12b–d,f display the density of SCs (0.1–5.0 μm) and the distribution of SCs (0.1–1.0 μm) in Q-T, D_5min_-T, D_5h_-T, and D_12h_-T samples. As most researchers reported [39,40,41], the density of SCs increases with the DCT processing time. In addition, the density of SCs has a significant improvement when the DCT time increases from 0 to 5 h. The increase in dislocation and the fine carbide precipitation during DCT promote the precipitation of SCs during tempering. However, the value of SC density in D_5h_-T and D_12h_-T is similar because the difference in microstructure (M and carbides) between D_5h_ and D_12h_ is slight. The densities of SSCs (0.1–0.5 μm) in Q-T, D_5min_-T D_5h_-T, and D_12h_-T are 6.14 × 10^10^ m^−2^, 6.48 × 10^10^ m^−2^, 7.05 × 10^10^ m^−2^, and 7.15 × 10^10^ m^−2^, respectively. The densities of MSCs in Q-T, D_5min_-T, D_5h_-T, and D_12h_-T are 8.78 × 10^10^ m^−2^, 9.01 × 10^10^ m^−2^, 9.15 × 10^10^ m^−2^, and 9.20 × 10^10^ m^−2^, respectively. Clearly, the rise in the SSCs is more dramatic than that in the MSCs. The densities of LSCs in Q-T, D_5min_-T D_5h_-T, and D_12h_-T are 3.64 × 10^10^ m^−2^, 3.70 × 10^10^ m^−2^, 3.81 × 10^10^ m^−2^, and 3.82 × 10^10^ m^−2^, respectively. The density value of the LSCs in all samples differs little. Figure 12f shows that the fractions of SSCs (0.1–0.2 μm) in D_5h_-T and D_12h_-T are higher by ~4% than those in Q and D_5min_-T. These results show that the increase in DCT processing time has a more obvious effect on the precipitation of fine SCs. Similarly, the fraction of SCs with a particle size of 0.6–0.7 μm in the tempered samples dominates the SCs (0.1–1.0 μm).

### 4.2. Effect of Martensite Multi-Level Microstructures on Mechanical Properties

According to the above results, the hardness of untempered samples that were deep- deep cryogenically treated for more than 5 h improved by ~44 HV_1_ more than the quenched sample due to the combined action of RA transformation (Figure 5) and dislocation increases (Figure 11). However, the hardness in the D_5h_-T sample only increased by ~23.8 HV_1_ more than that in the Q-T sample (Figure 2). Although the precipitation of fine SCs enhances the precipitation hardening process, the weakening of dislocation strengthening and the reduction in alloying elements in M leads to a decrease in hardness [22,42]. The increase in SC (0.1–5.0 μm) density with the DCT processing time simultaneously contributes to the homogeneity of hardness [18].

Previous studies reported that there is always an opposite relationship between hardness and impact toughness [12,43]. However, the impact toughness in D_5h_-T is ~32.4% higher than that in Q-T (Figure 2). The tempered samples exhibited a dramatic improvement in impact toughness, based on a few increases in hardness after the DCT process. The increase in SCs precipitation is one reason [44], while the evolution of M multi-level microstructures also plays a significant role.

M has a certain ductility compared with carbides [21], making a great contribution to impact toughness. Plenty of dislocation lines that are generated from vacancies and carbon atom segregation will occur during DCT, due to the lattice contraction of M and drastic temperature changes (Figure 6). The fresh MLs are formed with the dislocation lines as the boundary [22,45], which represents the refinement of M (Table 4). Meanwhile, the fresh M formed from RA is more refined than the original M.

The addition of dislocation lines and LAGBs during DCT promotes the generation of new martensitic boundaries (BBs and LBs). The formation of the new boundary contributes to the refinement of M multi-level microstructures, including MB and MP. Previous research showed that the cracks have large deflections at the martensitic boundaries, which means that the crack propagation is hindered by all the PBs, BBs, and LBs [22]. The increase in MB and MP can hinder the crack propagation path, which is more effective than ML [45]. Because both PB and BB boundaries are high-angle grain boundaries (HAGBs), HAGBs are high-energy boundaries and require more energy consumption to propagate across the boundary. Therefore, large deflections may occur at the PB and BB boundaries, while the crack propagation at the ML boundaries (LAGBs) mainly exhibits small deflections. The hindering effect of MB boundaries on crack propagation is similar to the MP boundaries, yet the size is far smaller than the pack size [22]. Meanwhile, LAGBs are less likely to cause stress concentrations and have fewer crack initiation points, due to their lower energy boundaries and stronger interfacial binding forces [26]. In addition, several investigations show that low-Σ CSL boundaries (e.g., Σ3) exhibit higher resistance to various types of intergranular degradation compared with random boundaries, enhancing the properties of the materials [35,46]. In conclusion, the refinement of M multi-level microstructures improves the impact toughness of tempered samples.

## 5. Conclusions

This study presents the evolution of martensitic multi-level microstructures for AISI M35 HSS that was deep cryogenically treated, with different DCT soaking times (0–48 h). Meanwhile, the effect of these microstructural variations on the mechanical properties (Vickers hardness and impact toughness) of the tempered samples was also discussed. The following conclusions can be derived:

1. The transformation of RA and the high plastic deformation that occurs during the DCT process increase the martensitic lattice strain and dislocations, reducing the temperature of carbide precipitation. The effect of DCT soaking time on the SCs precipitation of the DCT samples is limited. However, the increase in deep cryogenic time notably promotes the precipitation of fine carbides in tempered samples when the time is less than 5 h.
2. The rise in DCT processing time has a remarkable refinement of the martensite multi-level microstructures (including *d_b_* and *d_l_*) due to the formation of numerous fresh martensite and martensitic laths. However, DCT immersion time has a limited effect on the martensite multi-level microstructures when the DCT processing time is longer than 5 h. The addition of dislocations and LAGBs promotes the generation of new boundaries (BBs and LBs), contributing to the impact toughness of tempered samples.
3. The hardness of untempered samples that were deep- deep cryogenically treated for more than 5 h was ~900 HV_1_, improved by ~44 HV_1_ compared to the Q samples because of the transformation of RA and the increase in dislocation. The hardness in D_5h_-T is 867.05 HV_1_, which rose by ~22 HV_1_ more than that in Q-T, due to the precipitation of the SCs. Moreover, the impact toughness in D_5h_-T is 2.45 MJ·m^−2^, which is 32.4% higher than Q-T, owing to the refinement of M multi-level microstructures and the massive precipitation of SCs (especially the SSCs). When the DCT processing time is higher than 5 h, the increase in DCT immersion time has little effect on the other properties (Vickers hardness and impact toughness) of the selected steel.

## Figures and Tables

**Table 1:** Chemical composition of the AISI M35 high-speed steel.

Parameters	Element (wt %)								
	C	W	Mo	V	Cr	Co	S	P	Fe
Selected steel	0.92	5.97	5.13	1.84	3.82	5.00	0.03	0.03	Balance

**Table 2:** Different heat treatments and sample codes.

Groups	Detail of Treatment	Sample Codes
1	Quenching	Q
2	Quenching + tempering (550 °C × 2 h × 3)	Q-T
3	Quenching + DCT (x min/h)	D_xmin/h_
4	Quenching + DCT (x min/h) + tempering (550 °C × 2 h × 3)	D_xmin/h_-T

**Table 3:** The martensite tetragonality and carbon concentration.

Groups	Tetragonality (*c/a*)	Carbon Concentration (*C_c_*)/ wt %
Q	1.001482	0.032
D_5min_	1.004918	0.107
D_5h_	1.005337	0.115
D_12h_	1.005473	0.119

**Table 4:** The size of martensite block (*d_b_*) and martensite lath (*d_l_*) under different heat treatments.

Size/μm	Q	D_5min_	D_5h_	D_12h_
*d_b_*	1.135	1.106	1.028	1.021
*d_l_*	0.124	0.119	0.107	0.103

## Data Availability

The processed data required to reproduce these findings cannot be shared at this time, as the data also form part of an ongoing study.
